# Dysregulation in Retinal Para-Inflammation and Age-Related Retinal Degeneration in CCL2 or CCR2 Deficient Mice

**DOI:** 10.1371/journal.pone.0022818

**Published:** 2011-08-05

**Authors:** Mei Chen, John V. Forrester, Heping Xu

**Affiliations:** 1 Centre for Vision and Vascular Science, Queen's University Belfast, Belfast, United Kingdom; 2 Immunology and Infection, University of Aberdeen, Aberdeen, United Kingdom; Washington University, United States of America

## Abstract

We have shown previously that a para-inflammatory response exists at the retinal/choroidal interface in the aging eye; and this response plays an important role in maintaining retinal homeostasis under chronic stress conditions. We hypothesized that dysregulation of the para-inflammatory response may result in an overt pro-inflammatory response inducing retinal degeneration. In this study, we examined this hypothesis in mice deficient in chemokine CCL2 or its cognate receptor CCR2. CCL2- or CCR2-deficient mice developed retinal degenerative changes with age, characterized as retinal pigment epithelial (RPE) cell and photoreceptor cell death. Retinal cell death was associated with significantly more subretinal microglial accumulation and increased complement activation. In addition, monocytes from CCL2- or CCR2-deficient mice had reduced capacity for phagocytosis and chemotaxis, expressed less IL-10 but more iNOS, IL-12 and TNF-α when compared to monocytes from WT mice. Complement activation at the site of RPE cell death resulted in C3b/C3d but not C5b-9 deposition, indicating only partial activation of the complement pathway. Our results suggest that altered monocyte functions may convert the protective para-inflammatory response into an overtly harmful inflammation at the retina/choroidal interface in CCL2- or CCR2-deficient mice, leading to RPE and photoreceptor degeneration. These data support a concept whereby a protective para-inflammatory response relies upon a normally functioning innate immune system. If the innate immune system is deficient chronic stress may tip the balance towards an overt inflammatory response causing cell/tissue damage.

## Introduction

Age-related macular degeneration (AMD) is the leading cause of blindness in the elderly in developed countries. In the US in 2000, about 15% of people older than 80 years of age were estimated to have AMD [1] and this number is expected to increase by more than 50% by 2020, with over 2.95 million people having AMD [1]. In the UK, AMD accounts for almost 50% of those registered blind or partially sighted [2]. Clinically, AMD begins with drusen depositions between retinal pigment epithelial (RPE) cells and choroid at the macular region (so called age-related maculopathy, ARM). Visual acuity is normally not affected at this stage. The disease can develop into two forms causing substantial vision loss: “dry” AMD which refers to central geographic atrophy resulting from apoptosis of RPE cells, and subsequently the death of photoreceptors in the macula; and “wet” AMD which refers to neovascular or exudative AMD. Wet AMD is caused by the ingrowth of abnormal blood vessels from the choroid into the subretinal space leading to haemorrhage, leakage of fluid and eventual scar tissue formation and is usually associate with rapid vision loss. In contrast, dry AMD leads to photoreceptor and RPE cell atrophy with gradual visual loss. Treatments using anti-angiogenic biologic agents are available for wet AMD [3]–[5] but no specific therapy for dry AMD has been developed.

AMD is a multi-factorial disease. Aging is the primary disease determinant, while environmental factors such as smoking [6], [7], diet [8], [9], and light exposure are all risk factors of AMD [10]. Further, certain immune related gene polymorphisms also increase the risk of AMD [11]–[16]. Exactly how these multiple factors (aging, environment factors, and gene mutation) work together leading to AMD is not known. According to Harman's “free radical theory of aging” [17], age-related degeneration is an imbalance between free radical-induced tissue damage and the repair/remodelling process of the host. The immune system, in particular the innate immune system plays a crucial role in tissue repair/remodelling. It does so by mounting a para-inflammatory response [18]. We have shown recently that the para-inflammatory response in the aging retina consists of microglial activation and subretinal migration and complement activation [19]. The fact that polymorphisms of a number of immune related genes are risk factors of AMD led us to speculate that malfunction of the immune system may result in dysregulation of the para-inflammatory response in the aging retina, which may underlie the pathogenesis of AMD.

Previous studies have shown that mice with deletions of certain chemokine such as *ccl2* or chemokine receptors including *ccr2* [20] or *cx3cr1* [21] or both *ccl2/cx3cr1* [22] genes develop retinal lesions akin to human AMD. Chemokine and chemokine receptors are important for a normal immune function [23], [24]; these mice are useful models to address this question. In this study, we investigated the immune pathway(s) that might be dys-regulated leading to AMD-like changes in aged CCL2- or CCR2-deficient mice.

## Materials and Methods

### Mice

CCL2 KO mice (B6.129S4-*Ccl2^tm1Rol^*/J) and CCR2 KO mice (B6.129S4-*Ccr2^tm1Ifc^*/J) were originally purchased from the Jackson Laboratory (Bar Harbor, USA) and the wild-type C57BL/6J mice were provided by the Medical Research Facility of the University of Aberdeen. All mice were housed in a temperature and light controlled environment with a 12-h day-light cycle. The average light intensity inside the cage was 122 Lux. Regular health screens (quarterly) suggested that the housing environment was contaminated with mouse parvovirus and helicobacter but free of all other pathogens tested (a total of 13 mouse viruses, 11 bacterials/mycoplasma/fungi, 2 parasites were tested each time) over the period of this experiment. All animal procedures complied with and were carried out under the UK Animal Licence (Scientific Procedures) Act 1986, and were approved by the Ethical Review Committee of the University of Aberdeen.

### In vivo imaging

Mice were anaesthetised with a peritoneal injection of ketamine (60 mg/kg, Fort George Animal Centre, Southampton, UK) and xylazine (5 mg/kg, Pharmacia & Veterinary Products, Kiel, Germany). Pupils were dilated with 2.5% phenylephrine and 1% tropicamide (both from Chauvin, Essex, UK). Colour fundus images were obtained using the Topic Endoscopic Fundus Imaging (TEFI) system as previously described [25], [26]. Funds autofluorescent (AF) images were acquired using a home-built scanning laser ophthalmoscope (SLO) as described by us previously [27]. In our system a 1-mm beam with a 488 nm excitation from an argon laser (400 µW) was used to scan the fundus, AF signals were collected using a 520 nm long pass filter and stored as digital images with a resolution of 800×600 pixels. Fluorescein angiography (FLA) was performed using the same SLO setting and the detailed methods were described in our previous publications [28], [29]. In brief, mice were injected with 100 µl of 0.05% (v/v) sodium fluorescein (Sigma, Poole, UK) through the tail vein. A 488 nm excitation and 520 nm filter barrier were used to collect FLA images.

### Histopathology

Mouse eyes were carefully collected and fixed with 2.5% buffered glutaraldehyde (Fisher Scientific, Loughborough, UK) for at least 48 h. Samples were then embedded in paraffin wax for sections. Six sections (6 µm thickness) were cut from each eye at the level of or close to the optic disc, and the sections were stained using standard hematoxylin and eosin (H & E) methods. Images were captured using a light microscope (IMT-2, Olympus, Tokyo, Japan) fitted with a digital camera (Jenoptik Laser, Optik System GmbH, Germany). The number of the photoreceptors was counted in eight locations (four locations 600 µm apart at each side of the optic disc (see schematic picture in figure 2D)). At least three sections from each eye were counted and the averages were used to represent the eye.

For transmission electron microscopic (TEM) analysis, mice were perfused with 2.5% glutaraldehyde in PBS through the left ventricle under general anaesthesia. Eyes were removed and immersed in the same fixative for 48 h. After thorough washing, the anterior segment, lens and vitreous were removed. The posterior segment of the mouse eye was cut into small pieces and then post-fixed in osmium tetroxide, dehydrated in ethanol, and embedded in Epon. Ultrathin sections were stained with uranyl acetate and lead citrate, and examined with a Phillips CM 10 transmission electron microscope (Eindhoven, The Netherlands).

### Immunofluorescence staining

RPE-choroidal flatmounts were used to detect subretinal macrophages and the detailed method has been described previously [27]. The tissues were blocked and permeabilized with 5% bovine serum albumin (BSA) with 0.3% triton at room temperature for 1–2 h, and then incubated with primary antibody for 2 h followed by secondary antibody for a further hour. Primary antibodies used include goat anti-Iba-1 (1∶100, Abcam Ltd, Cambridge, UK), rabbit anti-arginase-1 (Santa Cruz Biotechnology Inc. California, LA, USA), goat anti-P2Y12 (Santa Cruz Biotechnology Inc.) and biotinylated rat anti mouse CD68 (AbD Serotec, Oxford, UK). Secondary antibodies used include FITC conjugated donkey anti-goat IgG (AbD Serotec), phycoerythrin (PE) conjugated anti-rabbit IgG (Invitrogen, Paisley, UK) and PE-streptavidin (BD Biosciences). At the end of incubation, samples were thoroughly washed and flatmounted in Vectashield (Vector Laboratory Ltd, Peterborough, UK) on glass slides with the RPE side face up. An LSM510 META confocal microscope (Carl Zeiss) was used for sample observation.

Cryosections of mouse eyes were immunostained to detect complement activation. Cryosections (9 µm) were fixed with 2% paraformaldehyde (Agar Scientific Ltd. Cambridge, UK) at 4°C for 30 min. After thorough washing, samples were blocked with 5% BSA for 20 min and then incubated with biotinylated anti-moue C3d (1∶100, R&D Systems, UK) and rabbit polyclonal anti-mouse C5b-9 ( 1∶100, Abcam Ltd, Cambridge, UK) for 1 h, followed by PE-conjugated streptavidin (1∶100, BD) and goat anti-rabbit FITC (Invitrogen, UK) for a further hour. Samples were mounted with Vectashield with DAPI and examined by confocal microscopy.

### Real-time PCR

Mice of different ages were sacrificed under CO_2_, eyes were collected and dissected immediately (within 5 min) and retinas were snap-frozen in liquid nitrogen. Total RNA was isolated using RNeasy Mini Kit (Qiagen, UK), and cDNA synthesized using SuperScript™ II Reserve Transcriptase (Invitrogen) with random primers (Invitrogen) following the manufactures' instruction. Real-time PCR was performed with the same amount of cDNA in LightCycler® 480 multiwell plate (384 wells) using Roche LightCycler® 480 SYBR Green I Master or Roche LightCycler® 480 Probe Master (Roche Diagnostics GmbH, Mannheim, Germany). C2, C3, C4, C5, CFI, TNF-α, iNOS, IL-10 and IL-12 were detected by SYBR Green method; CFB was detected by Taqman method using Universal Probe Library (UPL#1, Roche Diagnostics GmbH); CFH was detected by Taqman® gene expression assay (Assay ID: Mm01299243_m1, Applied Biosystems, California, USA). Primers were designed using web tool Primer 3 and synthesised by Invitrogen. Primer sequences are shown in table 1. Data were analysed using standard curve method. Relative expression of genes was normalised by housekeeping gene GAPDH.

**Table 1:** Primers used in real-time PCR.

Genes	Forward	Reverse
CFB	CTCGAACCTGCAGATCCAC	TCAAAGTCCTGCGGTCGT
C2	CTCATCCGCGTTTACTCCAT	TGTTCTGTTCGATGCTCAGG
C3	AGCAGGTCATCAAGTCAGGC	GATGTAGCTGGTGTTGGGCT
C4	ACCCCCTAAATAACCTGG	CCTCATGTATCCTTTTTGGA
C5	AGGGTACTTTGCCTGCTGAA	TGTGAAGGTGCTCTTGGATG
CFI	TTTCCCAACGAGTCTGTCCT	TGCAGTCCACCTCACCATTA
TNFa	GCCTCTTCTCATTCCTGCTT	CTCCTCCACTTGGTGGTTTG
iNOS	GGCAAACCCAAGGTCTACGTT	TCGCTCAAGTTCAGCTTGGT
IL10	TGCAGGACTTTAAGGGTTACTTGG	GGCCTTGTAGACACCTTGGTC
IL12	ATGGCCATGTGGGAGCTGGAGAAAG	GTGGAGCAGCAGATGTGAGTGGCT
GAPDH	ACTTTGTCAAGCTCATTTCC	TGCAGCGAACTTTATTGATG

### Cell culture

Mouse bone marrow-derived dendritic cells (BM-DCs) were cultured using a protocol originally described by Lutz and colleagues [30] and routinely used in our laboratory [31]. In brief, bone marrow cells were flushed from mouse femurs and red blood cells were removed with lysis buffer (Sigma). Cells were cultured on a microbiology culture dish in complete RPMI medium containing 20 ng/ml of GM-CSF (R&D). Medium was changed at day 3 and day 6. Eight days later, immature DCs were harvested from the culture dishes. Mature DCs were obtained by overnight incubating immature DCs with 1 µg/ml LPS (Sigma-Aldrich, UK). The phenotype of the cells were analysed by flow cytometry.

Primary bone marrow-derived macrophages (BM-DMs) were cultured as described^34^. Briefly, femoral bone marrow cells were isolated and cultured in 75 mm^2^ flasks in DMEM supplement with 10% heat-inactivated FBS plus 15% L929 supernatants, which served as a source of M-SCF. Medium was changed as require. Six days after, macrophages were collected for experiments. For phagocytosis assay, 1×10^5^ cells/well were seeded into 96-well plates. For cytokine expression, 5×10^5^ cells were seeded into 12 well plates, and activated with 1 µg/ml LPS for 8 h. Cells were then collected for total RNA isolation.

Mouse RPE cells were cultured using a previously described protocol [32]. Briefly, mouse eye cups were treated with 0.5% trypsin-EDTA for 30 min, RPE cells were harvested by aspiration and cultured in DMEM medium containing 10% FBS. Four days later, unattached cells were removed from the culture. RPE phenotype was confirmed by cytokeratin staining [32], [33]. Third-passage cells were used for the experiments described here. To collect RPE supernatants, untreated or 20 ng/ml TNF-α treated (24 h) RPE cells were washed with PBS and then incubated with serum-free culture medium for 12 h. The supernatants were collected and centrifuged at 800 g/min for 15 min and stored at −80°C for further experiment.

### Flow cytometry

Blood samples (100 µl), freshly isolated bone marrow cells and cultured BM-DCs, BM-DMs (1×10^5^) were washed with PBS (containing 0.5% BSA and 0.1% sodium azide) and then blocked with 10% normal mouse serum and 10% normal rat serum in PBS, followed by an incubation of FITC-, R-PE-, APC-, or PerCP-conjugated anti mouse CD11b, GR1, CD11c, CD86, F4/80, TLR2, TLR3, or TLR4 antibodies (all from BD Bioscience, Oxiford, UK) in dark for 30 min. After two washes with PBS, blood samples were incubated with FACS Lysing Solution (BD Bioscience) for 5 min. All samples were analysed by flow cytometry using CELLQuest Pro Software (Becton Dickinson, San Jose, CA, USA).

### Chemotaxis assay

Chemotaxis assays were performed using Transwell® (Costar, Corning Incorporated, Corning, New York, USA) in 24-well plates. Immature BM-DCs (5×10^4^ in 100 µl of RPMI1640) were seeded in the insert chamber (5.0 µm pore size, Costar). 200 µl serum-free medium containing different chemotaxins were added to the bottom chamber. After 2 h incubation (37°C in 5% CO2 incubator), the inserts were removed and the number of cells that had migrated to the bottom chamber were counted. To count the bottom chamber cells, five images were taken from the centre, left, right, superior and inferior fields of view of the chamber using an inverted microscope fitted with a digital camera (IMT-2, Olympus). The average number of cells from the five images of the same well was taken to represent the number of migrated cells per chamber well. All samples were studied in triplicate and the experiments were performed three times.

### Phagocytosis assay

Macrophage phagocytosis was conducted using the pHrodo™E. coli Bioparticles® Phagocytosis Kit (Invitrogen) following manufacture's instruction with slight modification. Briefly, 1×10^5^/well macrophages were seeded into 96-well plate and settled for 2 h before adding the same volume of bioparticles. Culture medium alone with bioparticles acted as negative control. Fluorescent intensity was measure at 30, 60, 120, and 180 min after introducing the bioparitcles with a fluorescence plate reader (excitation: 550 nm; emission: 600 nm). The net phagocytosis was calculated by subtracting the average fluorescence intensity of the no-cell negative-controls from the experimental wells.

### Endocytosis assay

Mannose receptor-dependent endocytosis was performed in immature BM-DCs and BM-DMs using lysine-fixable, anionic dextran fluorescein 40000MW (Molecular Probe, UK). Briefly, immature BM-DCs or BM-DMs (1×10^5^) were washed with PBS, and then incubated with 0.5 mg/ml of dextran fluorescein at 37°C for 90 min. Cells incubated with dextran on ice were used as background controls. At the end of the incubation, cells were washed with PBS and counterstained for CD11b, CD11c for DCs and CD11b, F4/80 for macrophages.

### Complement haemolytic assay

Blood was collected by cardiac puncture and serum was prepared immediately and stored at −80°C for the total complement activity measurement. Briefly, sheep erythrocytes (TCS microbiology, Claydon, UK) were first sensitized by incubating with rabbit anti-sheep erythrocyte stroma (Sigma-Aldrich) for 30 min at 37°C. Sensitized cells were washed three times in CFD (complement fixation diluents, Oxoid Ltd, Basingstoke, UK). 5×10^6^ sensitized erythrocytes were incubated for 30 min at 37°C with mouse serum. Then erythrocytes were centrifuged to form a pellet and the supernatant were collected for measurement of absorbance at 415 nm. Cells incubated in CFD only were used as negative controls (0% lysis), and cells incubated in dH2O as positive control (100% lysis).

### Induction of experimental autoimmune uveoretinitis (EAU)

We have shown previously that complement activation increases in the retina with EAU [34], [35]. We therefore, used EAU eyes as positive controls in the study of complement in the present work. EAU was induced in 8-week old female C57BL/6 mice with human interphotoreceptor retinoid-binding protein (IRBP) peptide 1–20 (Sigma) as previously described [25]. Previous studies have shown that complement activation is involved in EAU [35], [36]. Eyes from day 25 post-immunized EAU mice were used as positive controls for immunostaining of complement activation.

## Results

### Retinal degeneration in aged CCL2 KO and CCR2 KO mice

#### Clinical observations

**Figure 1:**
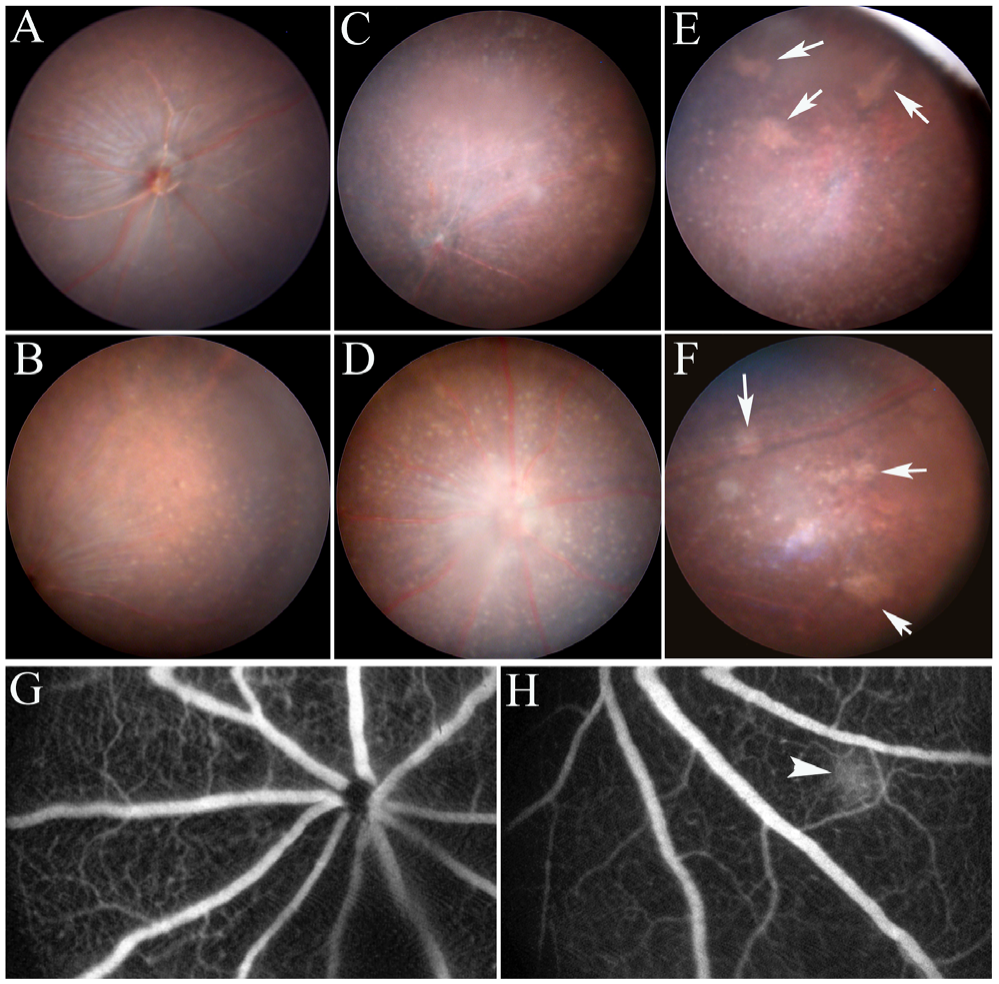
Fundus images of WT and CCL2 KO or CCR2 KO mice. (A–F) TEFI images of a 3-month old WT mouse (A), 24-month old WT mouse (B), 24-month old CCR2 mice (C, E) and 24-month old CCL2 KO mice (D, F). Arrows in E and F show patches of retinal lesion. G and H, fluorescein angiography images of a 3-month old WT mouse (G) and a 24-month old WT mouse (H). Arrowhead in H shows a localized fluorescein leakage.

Fundus observation revealed no abnormalities in young (3–6 months) WT, (Fig. 1A), CCL2- or CCR2-deficient (not shown) mice. Multiple yellowish “drusen-like” dots were frequently observed in the fundus of aged (18–24 months) WT (Fig. 1B), CCR2 KO (Figs. 1C, 1E) and CCL2 KO (Figs. 1D, 1F) mice. Although we were unable to accurately quantify the number of yellowish dots owing to developing cataract opacities, it appeared that more yellowish dots present in the fundus of aged CCR2 KO (Fig. 1C) and CCL2 KO (Fig. 1D) mice as compared with age-matched WT control mice (Fig. 1B) and this was further confirmed by confocal microscopy of RPE flatmounts (see below subretinal microglia section). Discrete areas of whitish lesions with ½∼2 optic disc in size were observed in 24% of CCR2 KO mice (15/62 mice, Fig. 1E, arrows) and 40% of CCL2 KO mice (22/54 mice, Fig. 1F, arrows) aged between 18–27 months, but not in any WT mice. Subretinal neovascular membrane was not observed in CCL2 KO, CCR2 KO, or WT mice of any age in fluorescein (Fig. 1G) or indocyanine green angiography. Occasionally, localised fluorescein leakage was observed in aged (>18 months) KO (1 out of 30 in CCL2 KO mice and 1 out of 32 in CCR2 KO mice) and WT mice (one out of 40, Fig. 1H, arrowhead), an indicator of the breakdown of the blood-retinal barrier (BRB) in aging animals reported previously in rats by Chan_Ling and colleagues [37].

#### Histology

**Figure 2:**
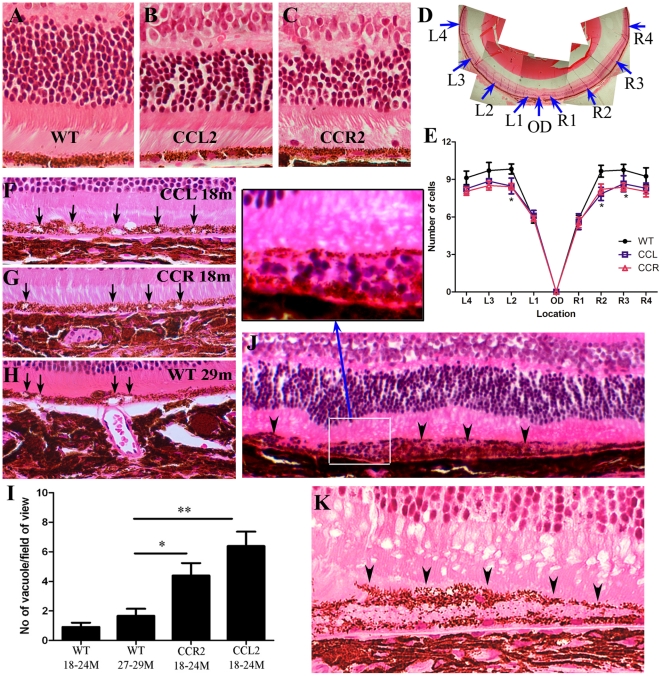
Histology of mouse eyes. Mouse eyes were taken from 18–24 months old mice and processed for H-E staining. A–C, representative images of a 22-month WT (A) a 22-month CCL2 KO (B) and a 22-month CCR2 KO (C) mouse showing photoreceptors. D & E, quantitative analysis of the number of photoreceptor nuclei. D, a schematic image showing the locations where the photoreceptor nuclei were counted. E, the numbers of photoreceptor in 20–24 months old mice WT, CCL2- or CCR2-deficient mice. *, P<0.05 compared to the WT mice at the same location . Mean ± SEM, n = 8–10 eyes, ANOVA (Kruskal-Wallis test) followed by Dunn's multiple comparison test. F–I, Representative images from aged CCL2 KO (F), CCR2 KO (G), and WT (H) mice showing RPE vacuolation (arrows). I, the number of RPE vacuoles in different strains of mice. Mean ± SEM, n = 12 eyes, *, P<0.05; **, P<0.01. ANOVA Dunn's multiple comparison test. J & K, retinal images from a 20-month (J) and a 24-month (K) CCL2 KO mouse showing areas of RPE cell damage (arrowheads) and inflammatory cell infiltration (insert in J). A layer of pigmented cells on top of degenerated (damaged) RPE cells was observed in K.

**Figure 3:**
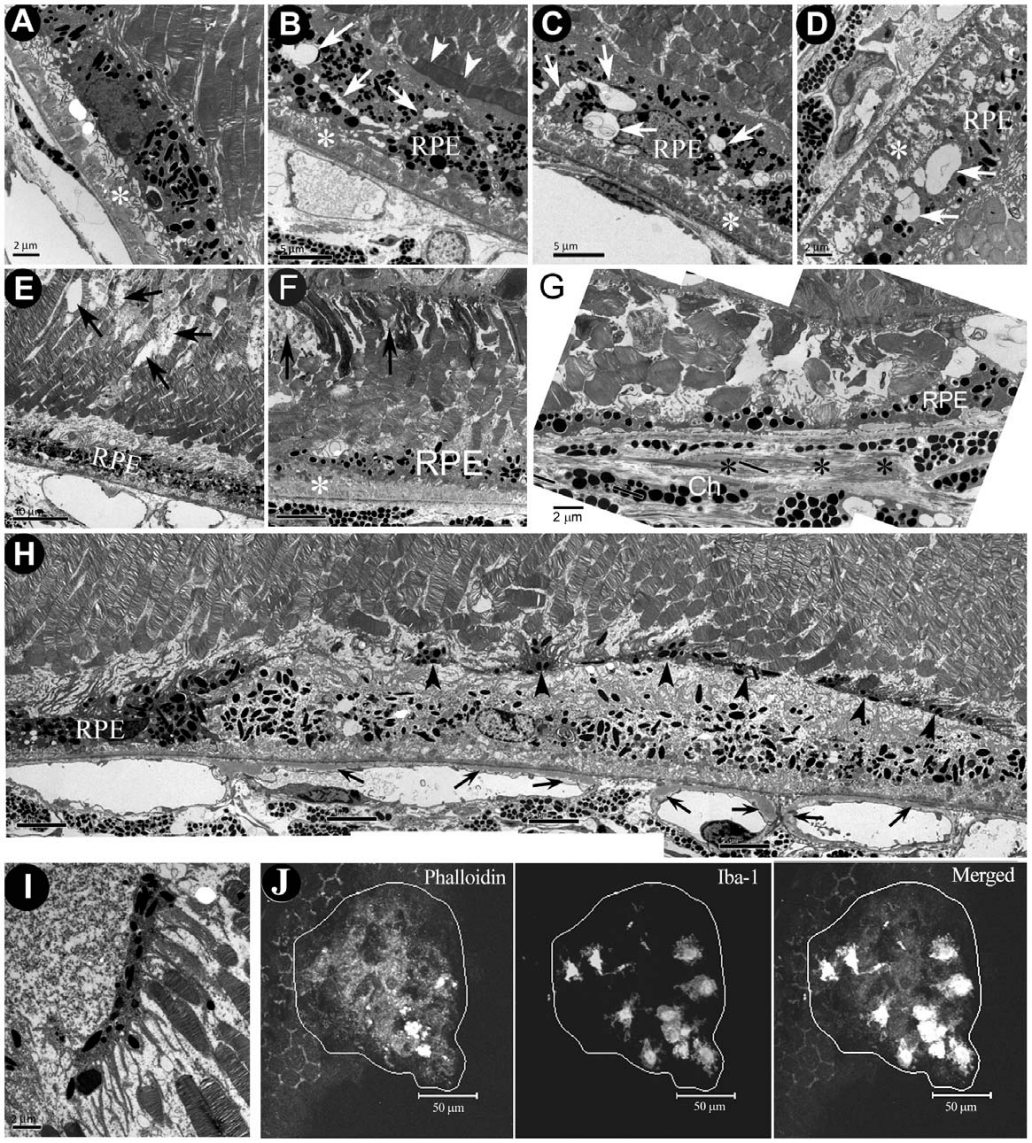
TEM images of RPE/photoreceptor of different strains of mice. A, an image from a 22-month old WT mouse showing RPE basal laminar deposits (asterisk). B, an image from 22-month old CCR2 KO mouse showing a photoreceptor outer segment (arrowhead) in parallel with RPE cells, multiple vacuoles (white arrows) in RPE cells and basal laminar deposits (asterisk). C, an image from a 22-month old CCL2 KO mouse showing multiple vacuoles in RPE cells (white arrows) and basal laminar deposits (asterisk). D, an image from a 24-month old CCL2 KO mouse showing multiple vacuoles and reduced melanin granules in degenerated RPE cells, basal laminar deposits (asterisk). E, an image from a 24-month old CCR2 KO mouse showing photoreceptor inner segment degeneration (black arrows), and a gap between RPE cells and photoreceptor outer segments. F, an image from a 20-month old CCL2 KO mouse showing photoreceptor inner segment degeneration (black arrows), reduced melanin granules in RPE cells and RPE basal laminar deposition (asterisk). G, an image from a 20-month old CCL2 KO mouse showing a patch of RPE atrophy, disorganised photoreceptor outer segments, a lack of choriocapillaris, and fibrotic tissues in the choroid (black asterisks). H and I, images from a 24-month old CCL2 KO mouse showing the loss of electron-dense materials in the cytoplasm, the loss of cytoplasm organelles and membrane, and a nucleus with reduced electron-dense materials in the RPE layer; choriocapillaris basal laminar deposition in the choroid; a layer of melanin granule-containing cells (block arrowheads) on top of the degenerated RPE cells with their process extending towards the photoreceptor outer segments. RPE, retinal pigment epithelia. (J), A confocal image of RPE/choroidal flatmount of a 24-month CCL2 KO mouse showing accumulation of macrophages/microglia at the site of RPE damage. RPE damages were highlighted with disorganised actins (phalloidin staining) and subretinal macrophages were labelled with Iba-1.

In H-E sections, no significant pathologies were observed in the retina of young mice of any strains (data not shown). In the aged mice (20–24 months old), the number of photoreceptor nucleus in the equator region was reduced in CCL2- or CCR2-deficient mice as compared to that in age-matched WT mice (Figs. 2A–E). Although “drusen-like” changes were observed on fundus examination (Fig. 1), typical drusens were not seen at the RPE/Bruch's membrane of any mice of any ages (Fig. 2). Vacuolated RPE cells (Figs. 2F–2H) were observed in aged (18∼24 months) CCL2 KO (Fig. 2F) and CCR2 KO mice (Fig. 2G). RPE vacuolation was also detected in very old WT mice (27∼29 months) (Fig. 2H). However, the number of vacuoles detected in RPE layer was significantly lower in aged WT mice than those in KO mice (Fig. 2I). Occasionally areas of RPE damage with inflammatory cell infiltration (Fig. 2J and insert) were detected in aged CCL2 KO mice. Sometimes a layer of melanin-containing cells placed on top of degenerating RPE cells with their process extending towards photoreceptors (Fig. 2K) was observed in aged CCL2 or CCR2 KO mice and was further confirmed by TEM (see Fig. 3H). Choroidal neovascularisation was not detected in any mice in histological examinations.

In TEM, although typical hard drusen was not detected in any strain of mice, basal laminar deposits were observed in both WT (Fig. 3A) and CCR2 KO (Fig. 3B) or CCL2 KO (Figs. 3C, 3D) mice of old age, which are consistent with soft drusen. RPE vacuolar changes were frequently seen in aged CCR2 KO mice and CCL2 KO mice (Figs. 3B–3D). Membranous residual material (autophagosome) was occasionally seen within the vacuoles (Fig. 3C). Disorganised photoreceptor outer segments (Fig. 3B, arrowheads) and gaps between photoreceptor outer segments and RPE cells (Fig. 3E) were frequently detected in CCL2- or CCR2-deficient mice. Further, degeneration of the photoreceptor inner segments was also observed in CCR2 (Fig. 3E) or CCL2 (Fig. 3F) deficient mice. Interestingly we detected two types of micrographic RPE damages in the KO mice. In the first type (Fig. 3G), RPE cells were extremely thin, and photoreceptor outer segments were totally disorganised. Underneath the thin RPE layer, choroid was devoid of choriocapillris and other vessels and replaced by fibroblast-like cells (Fig. 3G). It is possible that the RPE cells in this area had died and the thin melanin-containing structures were the extensions of neighbouring healthy RPE cells (in an attempt to restore the outer blood-retinal barrier). In the second type of damage (Fig. 3H), RPE cells have lost electron-dense materials, cell membranes, cell-cell junctions and many cytoplasmic organelles. Although the nuclear morphology was relative preserved, the electron-dense of the nucleoplasm was significantly reduced. These changes suggest that the RPE cells were undergoing early necrosis. In the choroid, there was a significant increase in the thickness of choriocapillaris basement membrane, particularly at the side adjacent to Bruch's membrane (arrows in Fig. 3H). On top of the degenerating RPE cells, a layer of melanin containing cells (presumably macrophages or microglial cells) with their processes extending towards the photoreceptor outer segments was observed (Figs. 3H, 3I). The accumulation of macrophage/microglia in the area of RPE cell damage in CCL2-deficient mice was further confirmed by confocal microscopy of RPE/choroidal flatmount (Fig. 3J).

### Subretinal microglia in aged CCL2 KO and CCR2 KO mice

**Figure 4:**
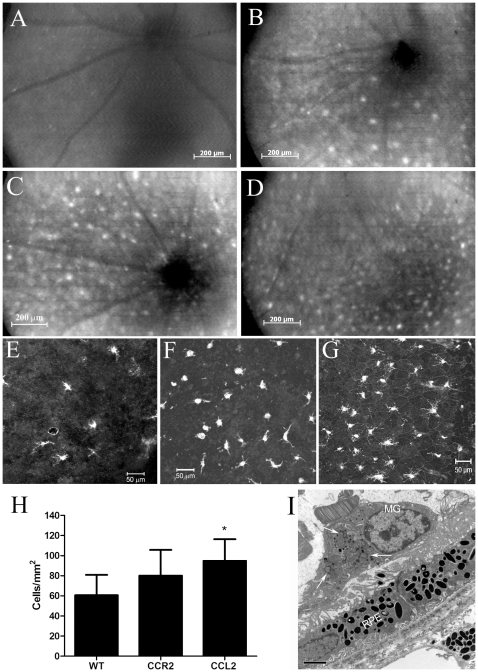
Subretinal microglia in different strains of mice. A–D, Autofluorescent (AF) images of a 3-month WT mouse (A), a 24-month WT mouse (B), a 24-month old CCR2 KO mouse, (C) and a 24-month CCL2 KO mouse (D). E–G, confocal images of RPE flatmounts stained for microglia with Iba-1 antibody (see Methods) from a 20-month old WT mouse (E), a 20-month old CCR2 KO mouse (F) and a 20-month old CCL2 KO mouse (G). (H) The number of subretinal Iba-1^+^ microglia in 20-month old of different strains of mice. Mean ± SD, N = 8∼12. *, P<0.05 compared to WT, ANOVA Dunn's multiple comparison test. I, a TEM image from a CCL2 KO mouse showing a subretinal microglial cell (MG) with lipofuscin (arrows) on the surface of RPE cells.

Previous studies have shown that “drusen-like” changes in aged CCL2 KO mice [38] and cx3cr1 KO mice [21] are subretinal macrophages. We have shown previously in wild-type C57BL/6 mice that fundus AF increases with age and lipofuscin-loaded subretinal microglial cells are the main sources of the fundus AF [27]. The similarity between the pattern of the fundus AF and yellowish dotted lesions suggests that these yellowish dots may be autofluorescent subretinal microglia. To test this hypothesis, fundus AF was performed in aged KO and WT mice followed by examination of RPE-choroidal flatmounts by confocal microscopy. Hyper-fluorescent AF was not seen in the fundus of young mice (Fig. 4A) but only in old mice (Fig. 4B–D). Many more hyper-fluorescent dots were observed in aged CCR2 KO (Fig. 4C) and CCL2 KO (Fig. 4D) mice as compared with that in age-matched WT controls (Fig. 4B) and this correlates to increased subretinal microglial (i.e. Iba-1^+^ cells) accumulation in the KO mice (Fig. 4E–H). Quantitative analysis of subretinal microglial cells in RPE-choroidal flatmounts revealed significantly more subretinal microglia in the CCL2 KO mice in comparison to age-matched WT controls. There were also more, but statistically non-significant numbers of subretinal microglial cells in the CCR2 KO mice (Fig. 4H). Transmission electron microscopy further confirmed lipofuscin granules in subretinal microglia (Fig. 4I arrows).

**Figure 5:**
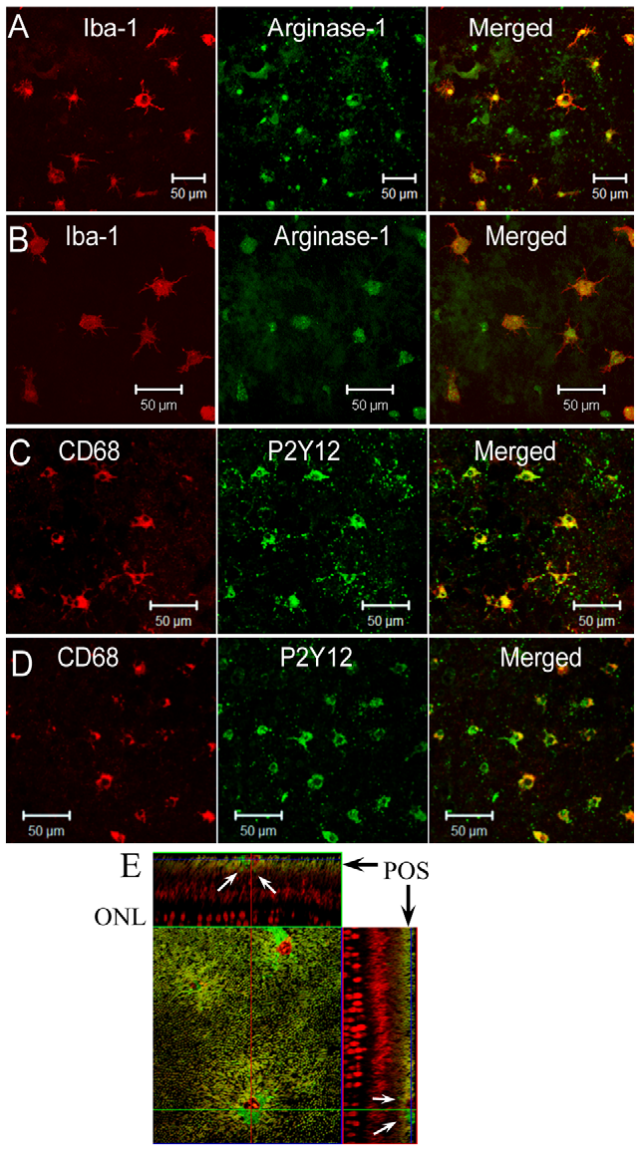
Phenotype of subretinal macrophage/microglia. RPE/choroidal flatmounts from aged (20–24 months) WT (A, C) and CCL2 KO (B, D) mice were dual stained for Iba-1/arginase-1 (A, B), or CD68/P2Y12 (C, D) and observed by confocal microscopy. Images presented are representatives from 6 mice in each group. E, Z-stack images of a retinal flatmount stained for Iba-1 (green) and Propidium iodide (PI) showing three Iba-1^+^ cells in the photoreceptor outer segment layer. Arrows: cell dendrites pointing toward the inner retina in z-sections.

We have shown previously that subretinal microglia are positive for Iba-1, CD68 and F4/80, but negative for MHC-II and CD206 [27]. In this study, we further examined the expression of arginase-1 and P2Y12 in these cells. Iba-1^+^ subretinal microglia were weakly positive for arginase-1 and strongly positive for P2Y12 in both WT (Figs. 5A, 5C) and CCL2 KO (Figs. 5B, 5D) mice. A small population (10–15%) of P2Y12^+^ cells were negative for CD68, suggesting a heterogeneous phenotype and possible subsets of subretinal microglia. In samples collected from 20∼24-month old mice, we did not detect any significant difference in the expression of CD68, arginase-1 and P2Y12 between WT (Figs. 5A, 5C) and CCL2 KO (Figs. 5B, 5D) or CCR2 KO mice (data not shown) (n = 6 mice in each group). Iba-1^+^ cells were also detected in the photoreceptor outer segment layer in retinal flatmount investigations (Fig. 5E). Dendrites of these cells can be seen pointing toward the inner retina in z-stack images (Fig. 5E, arrows).

### Complement activation in aged CCL2/CCR2 KO mice

Having shown that more RPE cell damage and more subretinal microglia existed in aged CCL2- or CCR2-deficient mice, we went on to investigate the mechanisms. Complement activation is believed to play a causal role in AMD [39]. Previously, we have shown that low levels of complement activation exist at the retinal/choroidal interface and complement activation increases with age [40]. Complement fragments C3a and C5a are strong immune activators and chemotactic factors for leukocytes and thus may induce microglia subretinal migration, whilst the membrane attack complex (C5b-9) may cause RPE or photoreceptor cell death.

**Figure 6:**
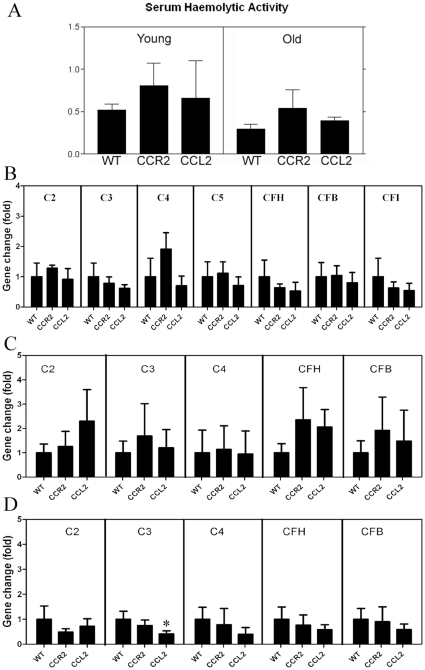
Complement expression in different strains of mice. A, Haemolytic activity of serum from different strains of mice. Mean ± SEM, N = 12 mice. B–D, complement gene expression in the liver (B), retina (C) and RPE/choroid (D) of different strains of 24-month old mice. Results are expressed as relative gene fold change of CCR2 KO or CCL2 KO mice against WT mice. Mean ± SEM, N = 6∼8, *, P<0.05 in comparison to WT mice, Dunnett's multiple comparison test.

Using a complement haemolysis assay, we found no significant difference between WT and CCL2 KO or CCR2 KO mice in both young (3 months) and old (24 months) groups (Fig. 6A), suggesting that serum complement activities between the WT and KO mice were the same. Furthermore, there was also no significant difference in the gene expression of complement components C2, C3, C4, C5, and complement regulatory factors CFH, CFI and CFB in the liver between WT and KO mice (24 months) (Fig. 6B).

Since complement components can also be produced locally in the eye [33], [33,39], we then compared the retinal (Fig. 6C) and RPE/choroidal (Fig. 6D) gene expression between WT and KO mice. The expression of complement C5 and CFI genes was below detectable levels in the retina and RPE/choroid using qPCR. CFH gene was slightly up-regulated in the retina of CCL2 KO mice (2-fold) compared with that of age-matched WT mice; however the difference was not statistically significant (Fig. 6C). There was no significant difference in the expression of other complement related genes (C2, C3, C4, CFB) between WT and KO mice in the retina (n = 6∼8) (Fig. 6C). In the RPE/choroid, reduced C3 expression was observed in CCL2 KO mice (Fig. 6D). No difference was observed in other genes between WT and KO mice (Fig. 6D).

**Figure 7:**
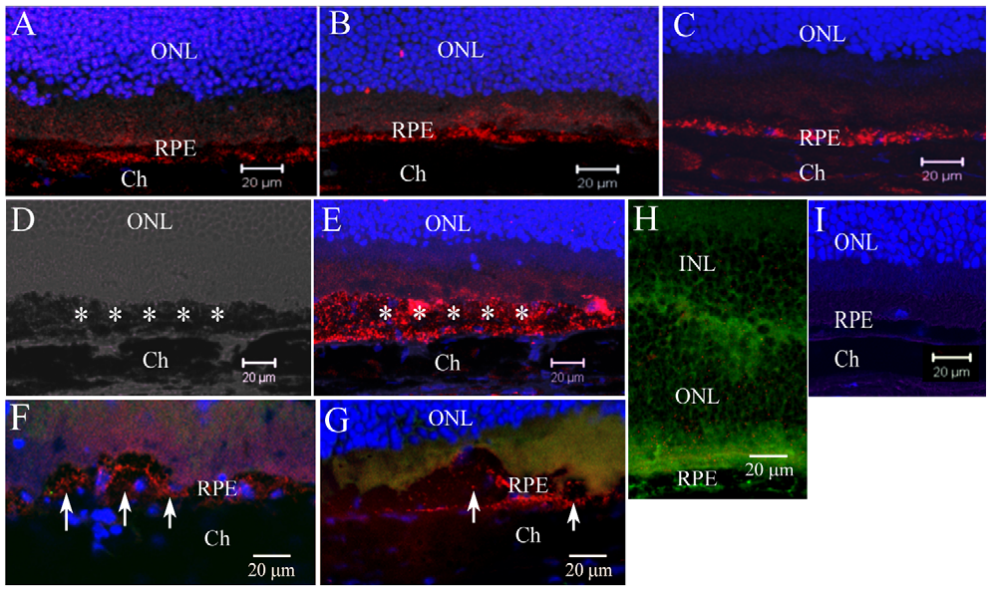
C3d and C5b-9 deposition at the retina/choroidal interface of different strains of mice. Cryosections of eyes from 22–24-month old mice were stained for C3d (red) or C5b-9 (green in F and G) and observed by confocal microscopy. A, an image from a 22-momth old WT mouse. B, an image from a 22-momth old CCR2 KO mouse. C, an image from a 22-momth old CCL2 KO mouse. D and E, images taken from a 24-momth old CCL2 KO mouse showing area of RPE cell death (asterisks) and extensive C3d deposition (red). F and G, images taken from a 24-momth old CCR2 KO mouse (F) and a 24-month old CCL2 KO mouse showing areas of RPE cell death (arrows) with C3d deposition (red), but no C5b-9 deposition (green). H, a retina from a mouse with EAU [25], [35] was used as positive control for C5b-9 staining (green). I, Isotype control antibody staining for C3d in a 24-month CCL2 KO mouse showing no background staining. ONL, outer nuclear layer. RPE, retinal pigment epithelia. Ch, choroid.

Previously, we have shown that complement C3d deposition at the retina/choroid interface increases with aged in WT mice [19], [40]. There was no significant difference in the amount of C3d depositions in the retina/choroidal interface between WT mice and KO mice (Figs. 7A–7C). However, increased C3d deposition was observed in the areas of RPE cell death in aged KO mice (Figs. 7D, 7E). Interestingly, the membrane attack complex (MAC) C5b-9 was not detected in the areas of RPE cell death in aged KO mice (Figs. 7F, 7G). Samples from inflamed EAU mice were stained positive for C5b-9 (Fig. 7H). Isotype control staining did not reveal any positivity in the sections (Fig. 7I). This result suggests that complement is partially activated at the retina/choroid interface in aged mice in response to RPE cell death and may be an attempt to clear the dead RPE cells.

### Monocytes/dendritic cells in CCL2 KO and CCR2 KO mice

**Figure 8:**
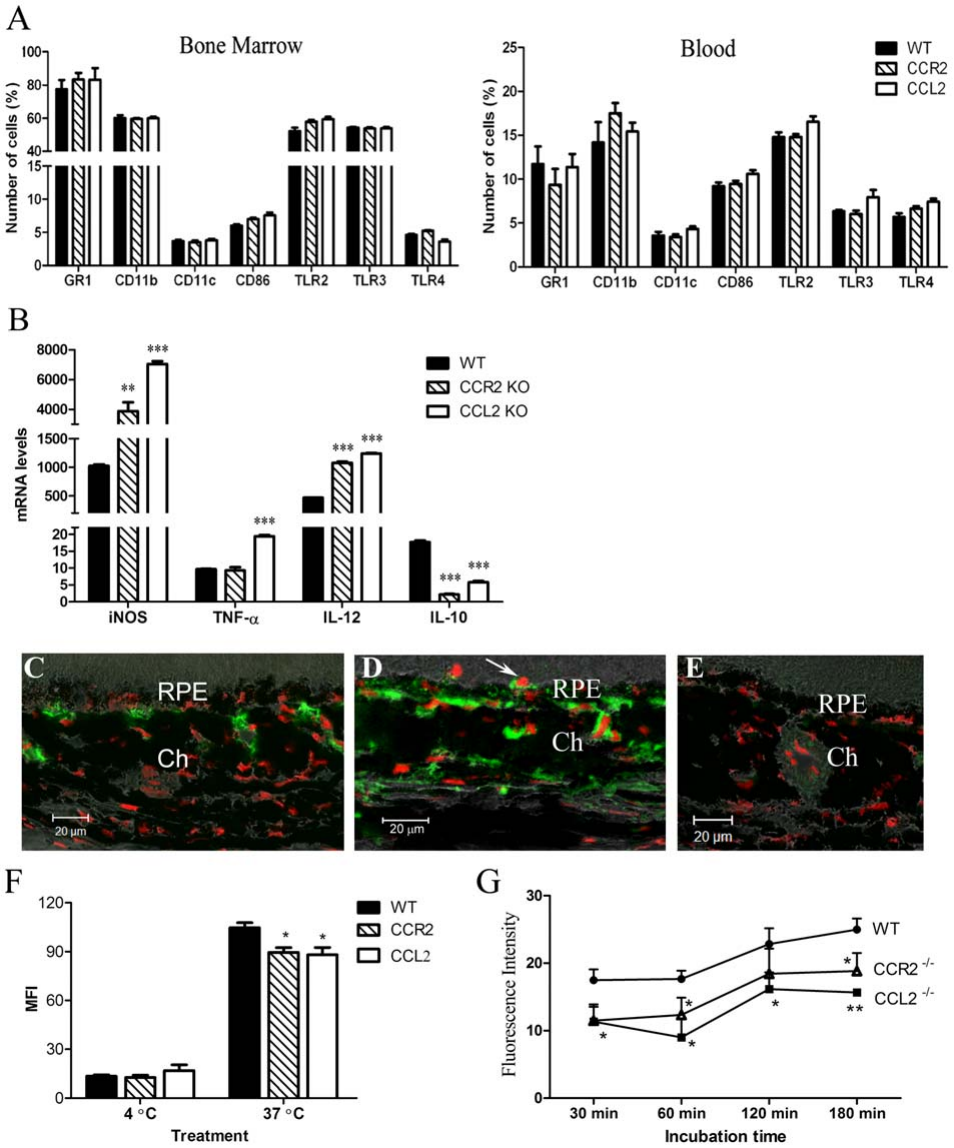
Phenotype and function of myeloid-derived cells in different strains of mice. A, Bone marrow and blood cells collected from different strains of mice were stained for different cell surface markers and analysed by flow cytometry. Mean ± SEM, N = 6. B, Cytokine gene expression in LPS stimulated BM-DMs of different strains of mice. Mean ± SEM, N = 3. *, P<0.05; **, P<0.01; compared to WT cells. Unpaired Student t test. Experiments were performed four times. C–D, Nitrotyrosine (green) and PI (red) staining in the eye of a 20-month old WT mouse (C) and a 20-month old CCL2 KO mouse (D). E, isotype control staining. Ch, choroid; RPE, retinal pigment epithelium. F, Endocytosis of BM-DMs using dextran method (see Materials & Methods). MFI, mean fluorescence intensity. G, Phagocytosis of E. coli by BM-DMs (see Materials & Methods). Mean ± SEM, N = 3. *, P<0.05, **, P<0.01 compared to WT cells of the same time point. Dunnett Multiple comparison test. Experiments were performed twice.

Macrophage activation/subretinal migration and complement activation are two major components of the para-inflammatory response in the aging retina [19]. Since complement activation is more likely to be the consequence rather than the cause of RPE cell death in aged CCL2- or CCR2-deficient mice, we then investigated the function of monocytes/macrophages. Flow cytometry did not reveal any significant difference in the phenotype of myeloid-derived cells in blood and bone marrow between WT and KO mice (Fig. 8A). BM-DMs from WT, CCR2 KO and CCL2 KO mice had similar phenotypes (data not shown). Unstimulated BM-DMs expressed low levels of iNOS, IL-12, TNF-a, and IL-10 genes and there was no significant difference on the expression levels between WT and KO mice (data not shown). The expression of these genes increased markedly (particularly iNOS and IL-12 genes) after LPS stimulation. LPS-stimulated BM-DMs from CCL2 KO and CCR2 KO mice expressed significantly more iNOS, IL-12, but less IL-10 as compared to their counterpart WT cells (Fig. 8B). BM-DMs from CCL2 KO mice also expressed significantly more TNF-α than WT cells (Fig. 8B).

To investigate whether the inflammatory phenotype of myeloid-derived cells exists in vivo in the eye, cryosections of mouse eyes were immunostained for nitrotyrosine, a product of tyrosine nitration mediated by reactive nitrogen species such as NO and peroxynitrite. Significantly more nitrotyrosine expression was detected in the choroid of aged CCL2-deficient mice (Fig. 8D) compared to age-matched WT mice (Fig. 8C). Subretinal macrophages/microglia were also positive for nitrotyrosine (arrow in Fig. 8D). Isotype control staining did not reveal any positivity (Fig. 8E).

Phagocytosis is one of the major functions of macrophages and dendritic cells. BM-DMs from CCL2 KO and CCR2 KO mice demonstrated reduced phagocytic function as determined by both dextran (endocytosis) (Fig. 8F) and E. Coli (phagocytosis) assays (Fig. 8G).

**Figure 9:**
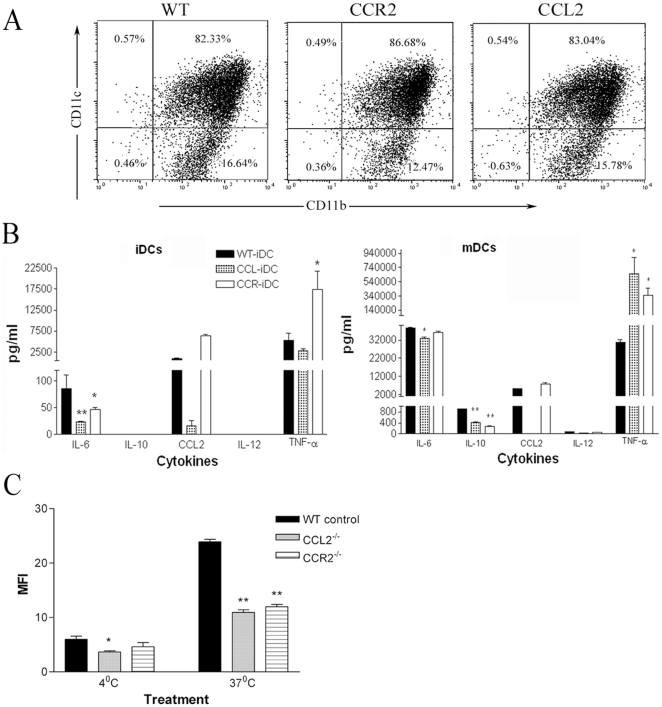
Phenotypes and functions of BM-DCs of different strains of mice. A, The expression of CD11b and CD11c in bone marrow-derived dendritic cells (BMDCs) of different strains of mice. B, cytokine production by immature BM-DCs (iDC) and mature BM-DCs (mDC) of different strains of mice. C, endocytosis of FITC-dextran by iDC of different strains of mice. Mean ± SEM, n = 4. *, P<0.05, **, P<0.01 in comparison to WT cells. n = 4, unpaired Student t test.

Dendritic cells are another major subset of myeloid derived cells. Bone marrow-derived dendritic cells (BM-DCs) from CCL2 KO or CCR2 KO mice had the same phenotype as those from WT mice (Fig. 9A). Similar to BM-DMs, BM-DCs (both immature and mature) from CCR2 KO or CCL2 KO mice produced significantly more TNF-α but less IL-10 than those from WT mice (Fig. 9B). BM-DCs from CCL2- or CCR2-deficient mice also have reduced endocytic activity when compared to WT BM-DCs (Fig. 9C).

### Chemotaxis of BM-DCs

**Figure 10:**
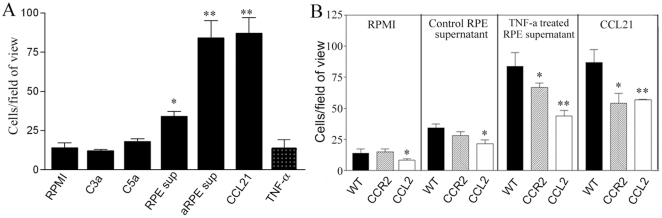
Chemotaxis of bone marrow-derived dendritic cells (BMDCs). A, migration of BMDCs of WT mice in response to different stimuli. *, P<0.05, **, P<0.01 in comparison to RPMI. Mean ± SEM, n = 3, Unpaired Student t test. B, the migration of BMDCs of different strains of mice in response to RPMI, RPE supernatant, TNF-a treated RPE supernatant and chemokine CCL21. *, P<0.05, **, P<0.01 in comparison to WT cells. Mean ± SEM, n = 3, Unpaired Student t test. Experiments were repeated twice.

To understand what might underlie the accumulation of subretinal microglia in aged mice, we carried out an in vitro chemotaxis assay using BM-DCs. Surprisingly, the complement fragments C3a and C5a did not induce BM-DCs migration (Fig. 10A), instead, RPE supernatant induced a significant level of BM-DC migration (Fig. 10A). Furthermore, the supernatant of TNF-α (20 ng/ml, for 24 h) treated RPE cells had even stronger chemotactic effect on BM-DCs in comparison to the supernatant from non-treated RPE cells, with an effect equivalent to 10^−8^ M of chemokine CCL21 (Fig. 10A). TNF-α alone did not induce BM-DC migration (Fig. 10A).

Having found that RPE supernatant, in particular TNF-αtreated RPE supernatant, was strongly chemotactic to BM-DCs, we then compared the chemotaxis of BM-DCs from different strains of mice. In the absence of any chemotactic factors (RPMI culture medium alone), there were small numbers of BM-DCs randomly migrated to the bottom chamber within 2 h (Fig. 10B). This random migration is reduced in BM-DC of CCL2 KO mice (Fig. 10B). In response to RPE supernatants (un-stimulated and TNF-α-stimulated) and the chemokine CCL21, BM-DCs from CCL2 KO mice migrated significantly less than cells from WT mice (Fig. 10B). BM-DCs from CCR2 KO mice showed a similar migratory ability as compared with WT cells in response to un-stimulated RPE supernatant (Fig. 10B); however, in response to TNF-α-stimulated RPE supernatant and CCL21, they migrated significantly less than WT cells (Fig. 10B). Thus while BM-DCs might be expected to be attracted to sites of damage or activated RPE cells, their ability to migrate from these sites may be impaired in CCL2/R2 mice and they would tend to accumulate at such sites.

## Discussion

Mice deficient in CCL2 or CCR2 were previously reported to develop a number of cardinal features of AMD when they aged, including drusen, photoreceptor atrophy and choroidal neovascularization (CNV) [20]. A more recent study by Luhmann and colleagues showed that “drusen”-like changes in aged CCL2-deficient mice were autofluorescent subretinal macrophages and the authors did not observe any geographic atrophy or CNV in these mice [38]. Using the same mice, in this study, we observed discrete patches of whitish retinal lesions in 40% of CCL2 KO mice and 24% of CCR2 KO mice (>18 month). Histological investigations revealed photoreceptor and RPE cell damage in aged KO mice, resembling early geographic atrophy in human AMD. However, similar to Luhmann et al's observation [38], we did not detect any CNV in these mice. We further showed that age-related atrophic retinal degeneration of the RPE and photoreceptors in these mice is unlikely to be caused by uncontrolled complement activation. Instead, we consider the partial activation of complement which we observed to be a consequence of RPE cell death in these mice and an attempt to remove the dead cellular material by apoptosis without overt inflammation (a state of para-inflammation [19]). Impaired migratory ability, reduced phagocytic function and excessive NO and TNF-α production in myeloid-derived cells in these genetically modified mice may alter the local para-inflammatory response resulting in more severe retinal degeneration.

The discrepancy in the degree of age-related degenerative retinal changes in these mice between our observation and those of others [20], [38] remains elusive. We believe that environmental factors are likely to be the major differentiating determinant. Light is one of the main factors that can induce retinal degeneration and microglia sub-retinal migration [41]. The mice in the present study were housed in a relatively bright environment (average illumination within the room was 500 Lux and within the cages was 122 Lux), which in this case is also assumed to have mouse parvovirus and helicobacter contaminations, but free of other pathogens. A recent study has shown that the retinas in CCL2 deficient mice are vulnerable to damage by chronic light exposure (Yu et al, IOVS, 2010, 51:E-abstract 5593). After 6 months chronic light exposure (9000 cd/m^2^, 2 h/day) patches of photoreceptor and RPE cell death were observed in CCL2 deficient but not WT mice (Yu et al, IOVS, 2010, 51:E-abstract 5593), which is in line with the current observation in these mice. As similar information (light exposure level in the animal house) was not available in previous two reports [20], [38], we are unable to conclude whether or not the animal housing condition is responsible for the occurrence of the disease in these mice. It is also worth pointing out, however, that only about 40% of aged CCL2 KO mice developed retinal lesion, and in Luhmann and colleagues study, in which the authors did not observe retinal pathology, clinical fundus examination was not performed [38]. It is possible that retinal lesions might have been missed in histological and electrophysiological investigations in the absence of clinical guidance. We are alerted to the possibility of these findings by the clinical fundus examination and thus were better places to seek these changes.

Nevertheless, the models allowed us to investigate the casual roles of monocyte malfunction (as a result of CCL2 or CCR2 depletion) in age-related retinal degeneration. The key pathology observed in aged CCL2 KO or CCR2 KO mice was the degeneration of RPE and photoreceptors. The number of the photoreceptors in aged CCL2 or CCR2 KO mice was significantly reduced in the equator area compared to age-matched WT control mice (Figs. 2A–2E); moreover, this effect correlated with increased RPE vacuolation and cell death in these mice (Figs. 2F–2I,and 3). Interestingly, in the area of RPE damage, we detected profound changes in the choriocapillaris including the increment in the basement membrane of choriocapillaris or the total loss of choriocapillaris. Whether the loss of choriocapillaris is the cause or consequence of RPE damage in this model remains elusive. Our observation, however, is in line with a recent study, which shows that the loss of choroidal circulation is related to retinal geographic lesion [42].

In line with the age-related RPE/photoreceptor degeneration in these mice, an innate immune activation at the retina/choroid interface, manifested as macrophage subretinal accumulation and complement activation (Figs. 4, 7), which was observed in aged WT mice (Figs. 4 and 7) [19], [27], [40], was enhanced in CCL2 KO and CCR2 KO mice. According to Medzhitov's “para-inflammation” theory [18], previously, we defined this low-grade chronic retina/choroidal inflammation as age-related retinal “para-inflammation” [19].

Of the two observed features of the age-related retinal para-inflammatory responses, i.e. macrophage/microglia subretinal migration and complement activation [19], CCL2- or CCR2- deficiency appeared to modify the macrophage/microglial response. Our examination of the complement system in these mice indicated that it seemed to function as well as in WT mice. Increased C3d but not C5b-9 deposition at the site of RPE cell death (Fig. 7) suggests that complement activation is the consequence rather than the cause of RPE damage. This result also suggests that complement activation at the retina/choroidal interface is tightly controlled. In the event of age-mediated RPE cell death, complement is partial activated (probably through the AP) to allow C3 to be cleaved without activating the terminal pathway. C3 cleavage generates C3 fragments (C3b/C3d), which can opsonise apoptotic cells promoting phagocytosis by macrophages.

Our major findings concerned the myeloid cell compartment. Monocytes from CCL2- or CCR2-deficient mice demonstrate reduced phagocytosis/endocytosis (Fig. 8) as well as impaired chemotaxis (Fig. 9), they also express enhanced iNOS, IL-12 and TNF-α but decreased IL-10 (Figs. 8, 9). These abnormal functions may alter the nature of macrophage/microglia para-inflammatory response. RPE cell supernatant, in particular from TNF-α stimulated RPE cells induces a significant number of BM-DC chemotactic response, suggesting that microglial cell subretinal migration in aging mice might be induced by soluble factors released by RPE cells, in particular RPE cells that were undergoing chronic stress. Once in the subretinal space, the role microglia might be assumed to remove/repair damaged cells/molecules and restore tissue homeostasis [19]. Cells in CCL2- or CCR2-deficient mice may not be able to do so as they have reduced phagocytic function; in fact, they may cause further damages to local tissue by producing large amounts of NO and TNF-α. Furthermore, such cells may have impaired ability to migrate away from the site of damage. Evidence of such a defect is suggested here in the experiments on the chemotactic response to CCL21. CCL21 is important for tissue DC homing to draining lymph nodes [43]. However, we show here that BM-DCs from CCL2- or CCR2-deficient mice have markedly reduced migratory ability in response to chemokine CCL21. Subretinal microglia/macrophages are believed to migrate away from the subretinal space to the uveal tissues [44], and hence presumably to the eye-draining lymph node and the spleen. Although the mechanism controlling microglial egress from the subretinal space is not known, our result suggests that subretinal microglia in CCL2- or CCR2-deficient mice are less able to migrate away from this site, and thus would result in increased subretinal microglial accumulation (Fig. 4). Prolonged exposure of microglia to the noxious microenvironment of the subretinal space in CCL2 KO or CCR2 KO mice may then convert the cells into a more aggressive phenotype, which may further damage RPE/photoreceptors by producing excessive NO and TNF-α.

In summary, we have shown in this study that aged CCL2 or CCR2 deficient mice develop certain features of atrophic, but not angiogenic AMD-like changes, and as such, may represent an animal model for early stage human geographic atrophy. More importantly, our data suggests that altered innate immune functions including excessive NO, IL-12 and TNF-α production by monocytes, reduced phagocytosis and impaired chemotaxis in these mice may result in a dysregulation in age-related retinal para-inflammatory responses, which may be responsible for the development of age-related atrophic retinal changes in these mice. Complement activation is unlikely to play a causative role in these models. Although a genetic study did not find any link between CCL2 and CCR2 gene polymorphisms and AMD risk [45], our data support the concept that malfunction in certain components of the innate immune system may lead to detrimental age-related immune responses instead of beneficial para-inflammatory responses, which may result in age-related retinal pathology. In addition, although complement did not seem to play a direct role in this model, a multifactorial pathogenesis is suggested by the combined effects of genetic myeloid cell dysfunction and the environmental risk of light damage, a condition highly reminiscent of the human condition.
